# Functions of Intracellular Alpha-Synuclein in Microglia: Implications for Parkinson’s Disease Risk

**DOI:** 10.3389/fncel.2021.759571

**Published:** 2021-10-04

**Authors:** Alix Booms, Gerhard A. Coetzee

**Affiliations:** Coetzee Laboratory, Department of Neurodegenerative Science, Van Andel Institute, Grand Rapids, MI, United States

**Keywords:** *SNCA*, alpha-synuclein, microglia, hematopoietic progenitors, differentiation, immune response

## Abstract

Alpha-synuclein accumulation in dopaminergic neurons is one of the primary features of Parkinson’s disease (PD). Despite its toxic properties during PD, alpha-synuclein has some important physiological functions. Although the activity of the protein has been extensively studied in neurons, the protein is also expressed in other cell types including immune cells and glia. Genetic studies show that mutations in synuclein alpha (*SNCA*), the gene that encodes alpha-synuclein, and alterations in its expression levels are a significant risk factor for PD, which likely impact the functions of a broad range of cell types. The consequences of altered *SNCA* expression in other cell types is beginning to be explored. Microglia, the primary macrophage population in the Central Nervous System (CNS), for example, are affected by variations in alpha-synuclein levels and functions. Studies suggest that deviations of alpha-synuclein’s normal activity influence hematopoiesis, the process that gives rise to microglia, and microglia’s immune functions. Alpha-synuclein levels also dictate the efficiency of SNARE-mediated vesicle formation, which could influence autophagy and cytokine release in microglia. Starting from the time of conception, these effects could impact one’s risk for developing PD. Further studies are needed to determine the physiological role of alpha-synuclein and how the protein is affected during PD in non-neuronal cells such as microglia. In this review we will discuss the known roles of alpha-synuclein in differentiation, immune responses, and vesicle formation, with insights into how abnormal alpha-synuclein expression and activity are linked to altered functions of microglia during PD.

## Introduction

Parkinson’s disease (PD) is characterized by the loss of dopaminergic neurons in the substantia nigra, leading to impaired motor function. Another pathological feature of the disease is the presence of intraneuronal aggregates of misfolded alpha-synuclein. Due to the complex ways in which disease risk is influenced by the interaction of genetics and environment, most PD is idiopathic and there are still no treatments to cure PD or slow its progression. Therapies targeting the protein alpha-synuclein, in efforts to reduce its aggregation within dopaminergic neurons, have, however, shown promising therapeutic potential (Brys et al., 2019; Cole et al., 2021). Despite the use of alpha-synuclein as a disease modifying target, there is still little known about its functions in a broad range of cell types and how the activity of the protein influences PD risk via such cell types.

A substantial body of research has focused on the role of alpha-synuclein in neurons, as its aggregation primarily occurs in, and is most highly expressed by this cell type (Bendor et al., 2013; Del Tredici and Braak, 2016). A disruption of alpha-synuclein’s normal function in other cell types, however, may contribute to PD initiation and/or progression. Genetic studies show that highly penetrant mutations, in addition to more common PD risk variants, affect the expression of synuclein alpha (*SNCA*) in PD (Chiba-Falek and Nussbaum, 2001; Farrer et al., 2004; Campelo and Silva, 2017). Cell types such as astrocytes, oligodendrocytes, microglia, and other immune cells do indeed express alpha-synuclein and are, thus, likely impacted by these genetic risk factors (Zhang et al., 2016; Reynolds et al., 2019). Microglia in particular have gained significant attention due to their involvement with prolonged CNS inflammation in correlation with alpha-synuclein aggregation and neurodegeneration in PD patients (Qian and Flood, 2008). Microglia release proinflammatory cytokines in the presence of exogenous alpha-synuclein when they become activated, and are one of the main cell types contributing to transmission of the misfolded protein between neurons (Sanchez-Guajardo et al., 2013). The extent to which microglia in PD patients show morphological and functional changes in correlation with alpha-synuclein aggregation has been extensively reviewed elsewhere (Ferreira and Romero-Ramos, 2018; Lecours et al., 2018). Most studies of microglia show that PD-associated genetic mutations give rise to abnormal microglia morphology (Lee et al., 2002; Su et al., 2009). However, these changes have been attributed to the interaction that microglia have with alpha-synuclein originating from other cell-types or outside sources. Importantly, such changes could be independent from those that are induced by exogenous alpha-synuclein mutant forms or aggregates, but this is still an emerging topic. Indeed there are studies showing functional changes in microglia as a result of endogenous *SNCA* expression changes (Grozdanov and Danzer, 2020). Investigating the function of endogenous alpha-synuclein in immune cell types, such as microglia, may reveal novel disease mechanisms involved in disease initiation and progression and provide additional strategies for therapeutic intervention. In this review we will discuss the known roles for alpha-synuclein with a focus on the relevance of its function in microglia during PD.

## Genetic Associations of *SNCA* With Parkinson’s Disease

*SNCA* was the first reported PD-associated gene to have a missense point mutation, resulting in an amino acid change from alanine to threonine at position 53 (A53T) in the alpha-synuclein protein (Polymeropoulos et al., 1997). Since then, four other mutations (A30P, H50Q, G51D, and E46K) have been found in patients with familial PD (Zarranz et al., 2004; Appel-Cresswell et al., 2013; Kiely et al., 2013; Rutherford et al., 2014). A triplication at the *SNCA* locus, leading to increased levels of alpha-synuclein, was also reported for autosomal dominant PD (Singleton et al., 2003; Miller et al., 2004). More recently, unbiased genome-wide association studies (GWAS) have identified common risk variants/single nucleotide polymorphisms (SNPs) in non-coding and coding regions in and around SNCA, that are associated with sporadic PD (Billingsley et al., 2018; Nalls et al., 2019). For example, the most highly significant PD-associated SNP, rs356182 (meta *p* = 1.85 × 10^–82^), determined in the latest metanalysis of PD, is located within an enhancer at the *SNCA* locus (Nalls et al., 2019). Interestingly, the protective allele (A) is associated with increased expression of *SNCA*, whereas the allele that carries the risk (G) is linked to lower expression levels of *SNCA* in cerebellar and substantia nigra tissue (Cooper et al., 2017). This SNP is situated in an enhancer that has pleiotropic consequences in neurons (Prahl et al., 2020). The data suggests that higher and lower levels of alpha-synuclein are associated with PD. In line with this complexity, eQTL (expression quantitative trait loci) data from GTeX (Genotype Tissue Expression Project) also shows that rs356182 has allele-dependent expression of *SNCA*, which is increased in some brain tissues and decreased in others. These results are derived from bulk tissue, making it difficult to interpret which cell types are truly affected by allelic differences. Taken together, the genetic data suggest a strong correlation between increased and decreased expression of *SNCA* leading to elevated risk for PD. However, the mechanisms and the cell types influenced by these genetic associations remain to be explored.

### Genetic Link Between Parkinson’s Disease and *SNCA* in Microglia

**FIGURE 1:**
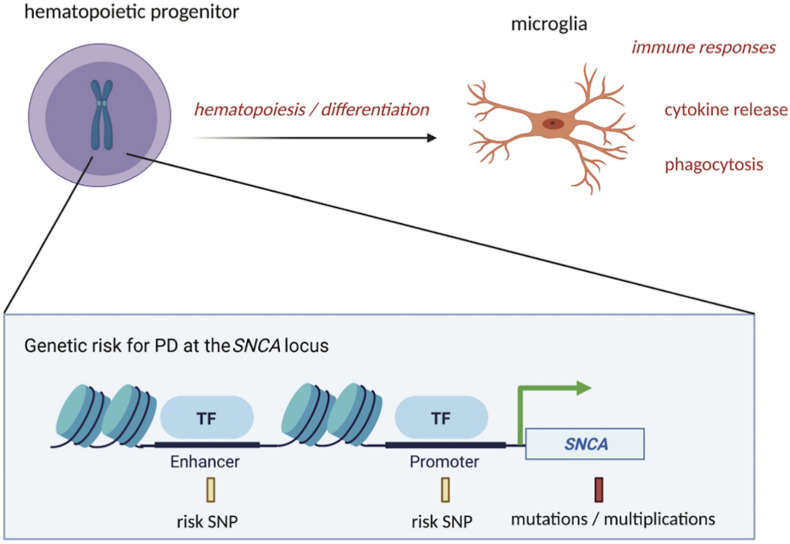
PD-associated genetic variation at the *SNCA* locus could affect microglia function at the precursor and mature stages. PD risk SNPs in regulatory regions of DNA, such as enhancers and promoters at the *SNCA* locus, along with more highly penetrant mutations or multiplications in *SNCA* coding regions, could impact the process of differentiation of hematopoietic precursors into mature microglia. Once differentiated, microglia’s functions, such as cytokine release and phagocytosis, could also be influenced by these genetic risk factors, contributing to initiation or progression of PD starting from the time of conception. This figure was created with BioRender.com.

Genetic risk may in part dictate alterations in pathways that influence microglia’s functions or abundance in the brain (Figure 1). For example, GWAS support a role for inflammatory pathways, like those involved in cytokine-mediated signaling, in PD susceptibility (Durrenberger et al., 2012; Holmans et al., 2013). Genes highly expressed in microglia, including *LRRK2* and lysosomal genes, also show enrichment of PD genetic risk variants (in or around the genes) that affect their expression levels (Reynolds et al., 2019). Genetic risk is, however, complex and although PD has been tied to genes, like *LRRK2*, the complete set of genes in microglia and their functions that lead to PD susceptibility are only beginning to be explored. In a previous study (Booms et al., 2020), we searched for PD risk SNPs that reside in regulatory DNA in microglia and found one such SNP (rs2737004—highlighted in yellow in Supplementary Table 1) located in an intergenic enhancer at *SNCA.* The full list of SNPs at the SNCA locus can also be found in Supplementary Table 1. We believe that rs2737004 is exerting its effects through the regulation of *SNCA* expression. Although in general, *SNCA* is known to be lowly expressed in microglia, our study indicates that a portion of PD risk may be acting through this genetic variant, which could influence *SNCA* expression in microglia. Indeed, studies (discussed below) that modulate *SNCA* expression in microglia show an effect on their functions (Grozdanov and Danzer, 2020). Furthermore, few studies have evaluated *SNCA* expression in microglia depending on maturation or activation status (also discussed below). We therefore argue that more emphasis should be placed on examining the mechanisms in microglia involving the regulation of *SNCA* dictated by genetic risk variants or more highly penetrant mutations. Figure 1 outlines a general depiction of genetic risk at the SNCA locus. To further dissect the link between genetic risk for PD and alterations in *SNCA* expression, it will be important to continue to uncover the full scope of alpha-synuclein functions in cell types such as microglia in addition to neurons.

## Known Functions of Alpha-Synuclein

The molecular functions of alpha-synuclein are still incompletely understood, but studies investigating its activity have shown that under normal conditions it facilitates the release and transport of dopamine, promotes fibrilization of microtubule associated protein tau (MAPT), and prevents caspase-3 activation through p53 inhibition to protect against cell death (da Costa et al., 2000; Alves Da Costa et al., 2002; Siddiqui et al., 2016). In PD, *SNCA* adopts more neurotoxic forms, especially due to mutations, such as A53T, that promote its aggregation (de Oliveira and Silva, 2019). These findings have been primarily demonstrated in neuronal cells. However, alpha-synuclein is also involved in processes regulating differentiation, induction of inflammatory responses, and SNARE-mediated vesicle formation, which may impact the behavior of other non-neuronal cell types. These studies are discussed below.

### Alpha-Synuclein in Differentiation

Multiple studies provide evidence that alpha-synuclein plays a key role in differentiation. For example, erythrocytes express a substantial amount of alpha-synuclein, with expression increasing during terminal differentiation to promote protein enucleation of erythroblasts and stabilize erythroid membranes (Satoh and Kuroda, 2001; Kim et al., 2018). In the MG63 osteosarcoma cell line, overexpression of alpha-synuclein caused these cells to adopt a more differentiated phenotype (Huang et al., 2017). Furthermore, NTera2 teratocarcinoma cells showed an increase in alpha-synuclein expression levels during neuronal differentiation (Kuure et al., 2007). Barbour et al. (2008) also demonstrated that alpha-synuclein affects neuronal differentiation. Overexpression of wild-type or mutant A53T alpha-synuclein interfered with retinoic acid-induced differentiation in SH-SY5Y cells, indicating that PD-associated mutations may also influence the normal differentiation process. These cells had lower levels of tyrosine hydroxylase and dopamine transporter, that correlated with a reduction in neurite outgrowth compared to control cells. Results from this study were also confirmed in murine primary cultured mesencephalic dopaminergic neurons expressing A53T mutant alpha-synuclein. Interestingly, the mechanism leading to the loss of the differentiation phenotype was through interference with GSK-3β/β-catenin signaling, a key pathway in neurogenesis and differentiation of other cell types and fetal tissues, such as the mesoderm where microglia originate (Fujita et al., 2007; Araki et al., 2018). In line with these observations, iPSC-derived neurons from a PD patient with an *SNCA* triplication had a reduced capacity to differentiate into dopaminergic and GABAergic neurons (Oliveira et al., 2015). Multiple studies, thus indicate that differentiation is affected by changes in alpha-synuclein that could potentially influence PD risk.

### Alpha-Synuclein and Hematopoiesis/Differentiation in Microglia

Microglia originate from yolk sac-primitive macrophages and colonize the brain at a very early stage in embryonic development as a part of “primitive hematopoiesis.” Hematopoietic stem cells generated during “definitive hematopoiesis” migrate from the yolk-sac to the fetal liver and bone marrow, where they then differentiate into monocytes, macrophages, and lymphocytes (Alliot et al., 1999; Ginhoux et al., 2010; Kierdorf et al., 2013; Ginhoux and Prinz, 2015). Although microglia and other immune cell lineages arise during two distinct hematopoietic processes, cells like macrophages, monocytes, and dendritic cells are classified as mononuclear phagocytes, along with microglia, based on common cell surface markers, functions, and the possibility that they are derived from a common early hematopoietic precursor (Ransohoff and Cardona, 2010). There may, therefore, be similarities between microglia and peripheral monocyte hematopoietic precursor functions.

In addition to the cell types discussed above, some evidence suggests that alpha-synuclein plays a prominent role in differentiation of cells derived from the hematopoietic lineage (Pei and Maitta, 2019). *SNCA* expression is relatively strong in peripheral hematopoietic precursor cells and mature erythrocytes, with knockout of *SNCA* in mice leading to dysfunctional hematopoiesis (Tashkandi et al., 2018; Grozdanov and Danzer, 2020). Furthermore, Xiao et al. (2014) showed that alpha-synuclein is important during late hematopoiesis and B cell lymphopoiesis, suggesting a role for alpha-synuclein in differentiation of immune cells. Moreover, abnormalities in hematopoiesis have been observed in PD (Savica et al., 2009; Pei and Maitta, 2019). For example, PD patients who experienced anemia early in life were more likely to develop PD later in life (Kasten et al., 2010). In addition, a greater percentage of monocyte precursors were observed in the blood of PD patients, suggesting that these cells may not properly differentiate (Funk et al., 2013). The involvement of *SNCA* in hematopoiesis is still elusive. However, low levels of *SNCA* transcripts were found in PD patient’s blood samples and correlated with cognitive decline (Locascio et al., 2015). Animal studies demonstrate the involvement of *SNCA* in differentiation of cells derived from the hematopoietic lineage and human studies show that disruption in hematopoiesis is linked to PD. Therefore, more emphasis should be placed on the investigation of hematopoietic changes linked to *SNCA* expression abnormalities as this may be an early event that drives disease initiation.

**FIGURE 2:**
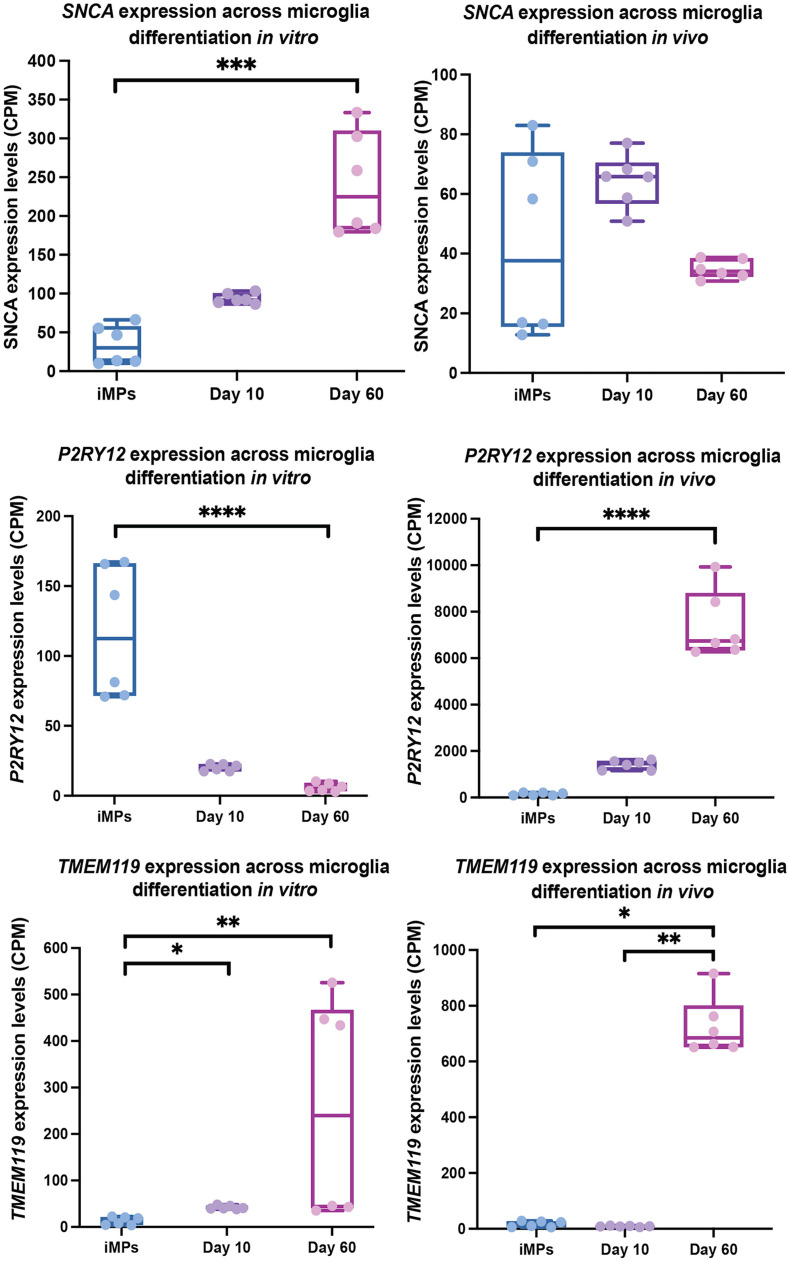
*SNCA, P2RY12, and TMEM119* expression in microglia precursors across differentiation *in vivo* and *in vitro.* This data is a re-analysis of publicly available data from Svoboda et al. (2019). CPM normalized expression levels of *SNCA, P2RY12, and TMEM119* are shown for microglial iMPs (induced microglia precursors) that have been transplanted into mice and then differentiation *in vivo* (left) or differentiated *in vitro* (right) for 10 and 60 days. Significance between timepoints was determined using a Kruskal-Wallis test with Dunn’s Multiple Comparison (^∗^*p* ≤ 0.05, ^∗∗^*p* ≤ 0.01, ^∗∗∗^*p* ≤ 0.001, ^****^p ≤ 0.0001).

Microglia are derived through the hematopoietic lineage and display many of the same immune properties as peripheral monocytes and macrophages, but the role of alpha-synuclein in the differentiation of these cells has not yet been explored. Microglia can self-renew and undergo mitosis to increase their numbers in the affected area during insult (Ajami et al., 2007). Therefore, the capacity of these cells to differentiate is critical to maintain the health of the CNS. Differentiation of microglia during primitive hematopoiesis at the time of embryogenesis could also affect the number of mature microglia residing in the CNS at the time of birth. The evidence showing that *SNCA* is expressed at high levels in hematopoietic precursors and influences the function of the hematopoietic system, which gives rise to immune cells, provokes the question of whether alpha-synuclein influences differentiation of microglia. RNA-seq data from mice indicates that *Snca* is expressed at higher levels in embryonic microglia compared to early post-natal and more mature microglia (Li et al., 2019). It is unknown, however, if *SNCA* is expressed in microglia precursors in humans and how that expression changes with differentiation. We thus evaluated existing data sets to determine if *SNCA* is expressed at higher levels in microglia precursors compared to cells that are further along the differentiation process into mature microglia (Figure 2).

To evaluate expression of *SNCA* in microglia precursors and throughout differentiation, we analyzed *previously published* data from Svoboda et al. (2019), who conducted a study using immature hiPSC-derived microglia that were either cultured *in vitro* or transplanted into mice at the precursor stage (iMP). They performed bulk RNA-seq of *in vivo* cells at 10- and 60-days post implantation and in *in vitro* cultured cells at 10- and 60- days of differentiation. After examining this data set for *SNCA* expression in microglia precursors compared to more mature *in vivo* and *in vitro* microglia, the data showed changes in *SNCA* across maturation time points. TMM normalized RNA-seq counts were derived using edgeR, and the significance between timepoints was determined using a Kruskal-Wallis test with Dunn’s Multiple Comparison to account for some of the within group variation (Figure 2). In line with what Svoboda et al. (2019) showed, the data demonstrates that *SNCA* expression significantly increased after 60 days cultured *in vitro*, whereas *SNCA* expression remained relatively similar across time points *in vivo*.

The data confirm that *SNCA* is expressed in microglia precursors, although the pattern across differentiation is different between *in vivo and in vitro* conditions. We observed a general pattern in *SNCA* expression for *in vivo* microglia, where *SNCA* expression is highest prior to or at the start of differentiation (day 10), with levels decreasing as the cells mature (Figure 2). However, the difference was not significant. This pattern is like what is seen in other cell types. For example, *in vitro*, dividing oligodendrocyte precursors show high *SNCA* expression and alpha-synuclein levels, which are lost during maturation (Djelloul et al., 2015). This is contradictory to what is seen in neurons where alpha-synuclein levels increase upon maturation (Pierce et al., 2018). These results highlight a possible role for alpha-synuclein in differentiation in non-neuronal cell types, whereas in neurons, in addition to differentiation, alpha-synuclein facilitates neuronal specific processes like synaptic transmission following maturation. Although the *in vivo* data in microglia for *SNCA* expression across timepoints is inconclusive, a more extensive evaluation in human microglia, by conducting a trajectory analysis using single-cell data from the human brain, could give better insights into the pattern of *SNCA* expression during the transition from hematopoietic stem cells to mature microglia.

An intriguing observation from this data is that *SNCA* expression increases throughout differentiation *in vitro*. Although this could be a factor of differentiation conditions, it is unknown whether the differences between *in vivo* and *in vitro SNCA* expression across time is related to activation status. Using single-cell data, the authors showed that iMG differentiated *in vitro*, more closely represent cells in a disease state than microglia differentiated *in vivo*, which could indicate that they are more activated. Indeed, it has been shown that microglia in culture adopt a more activated state and tend to lose expression of microglia specific markers, such as *P2RY12* and *TMEM119* (Butovsky et al., 2014; Bohlen et al., 2017; Gosselin et al., 2017). Svoboda et al. (2019)’s data, indicates this to be partially true, as *P2RY12* expression decrease over the course of differentiation *in vitro*, but increased over time *in vivo*. Whereas *TMEM119* expression increased by day 60 in both conditions (Figure 2). However, the data points for expression of *TMEM119* at day 60 in the *in vitro* group appear to have a large bimodal distribution. With having no prior knowledge on the necessary experimental design for each bulk RNA-seq sample, we attempted to do a batch correction using RUVseq, which helps to remove unknown sources of unwanted variation (Risso et al., 2014). Still, the bimodal distribution remained. We thus interpret these results with caution. Based on this data, the relationship between *SNCA* expression and microglia activation state is still unclear. Further studies are needed to explore the role of *SNCA* in microglia precursors and activated microglia to determine if alpha-synuclein influences differentiation or changes with activation state under physiological or disease conditions.

### Alpha-Synuclein’s Role in Immune Cell Recruitment and Inflammation

Infections from bacterial and viral pathogens are associated with high risk for PD. Patients with PD have been observed to have altered gut microbiota that consists of increases in certain types of bacteria such as *Lactobacillus* and decreases in others such as *Prevotella* (Scheperjans, 2018; Johnson et al., 2019). There is also a causative relationship between viruses like influenza, Herpes Simplex Virus 1, Epstein Barr Virus, and others with the development of Parkinsonism symptoms (Limphaibool et al., 2019). These infections are thought to act as one of the initiating factors that, in combination with genetic and other environmental risk factors, leads to elevated alpha-synuclein levels.

Although alpha-synuclein is present in enteric nerves of both PD and healthy patients, it is speculated that inflammation associated with infection leads to increased alpha-synuclein aggregation (Johnson et al., 2019). Exposures to infection are indeed linked to changes in alpha-synuclein expression, which also correlate with immune cell recruitment (Labrie and Brundin, 2017). For example, alpha-synuclein expression increased in primary neurons following infection with West Nile virus (Beatman et al., 2015). In addition, alpha-synuclein knock-out mice had an increase of infectious viral particles that correlated with lower survival, indicating a role for alpha-synuclein in restricting infection (Beatman et al., 2015). Another study by Stolzenberg et al. (2017) showed elevated levels of alpha-synuclein in enteric neurites of the upper GI tract of pediatric patients with gastric and duodenal inflammation. Expression of alpha-synuclein in enteric neurons was also induced in an additional cohort of patients with confirmed cases of norovirus (Stolzenberg et al., 2017). In the same study, oligomeric forms of alpha-synuclein acted as chemoattractant for neutrophils and monocytes. Thus, convincing evidence indicates that alpha-synuclein has a role in early immune responses.

### Alpha-Synuclein’s Involvement in Immune Responses in Microglia

The early immune response involving alpha-synuclein is not restricted to neurons. Human macrophages in culture had increased levels of alpha-synuclein following exposure to LPS and IL-β, indicating that infection affects the levels of endogenous alpha-synuclein, which consequently influences macrophage function (Tanji et al., 2002). Microglia, the resident macrophages in the brain, are highly implicated in PD pathogenies through their immune response to exogenous alpha-synuclein, but there is evidence to suggest that alpha-synuclein expression within microglia influences their immune responses as well (Grozdanov and Danzer, 2020). One of the first studies demonstrating the effects of endogenous alpha-synuclein on microglia function was done in 2006 by Austin et al. (2011). They found that microglia isolated from *Snca*-/- mice had an increased reactive phenotype at baseline and following stimulation with LPS as compared to microglia from *Snca* wild-type mice. After stimulation, *Snca*-/- microglia also had elevated levels of cytokine release accompanying an impairment in phagocytosis. In drosophila, overexpression of wild-type alpha-synuclein under the control of a pan-glial promoter (*repo-GAL4*) led to alpha-synuclein aggregation, death of dopamine neurons, and locomotion and autonomic dysfunction that was worsened in combination with co-expression of alpha-synuclein in both glia and neurons (Olsen and Feany, 2019).

Although these experiments highlight the potential for alterations in *SNCA* expression or alpha-synuclein levels to lead to functional changes in microglia, most of these studies relied on episomal expression constructs or whole-body knock-out animal models, which may not mimic physiological conditions related to genetic alterations in microglia *SNCA* expression in humans. Haenseler et al. (2017), however, generated iPSC-derived macrophages from PD patients with *SNCA* A53T mutations or *SNCA* triplications. The A53T mutant cell lines showed no effects, whereas iPSC-derived macrophages from patients with an *SNCA* triplication showed elevated levels of alpha-synuclein and a decrease in phagocytosis and cytokine release. Although macrophages share similar functions to microglia, they may behave differently under normal conditions or during disease (Yamasaki et al., 2014; Grozdanov and Danzer, 2020). More work is needed to understand the influence of endogenous *SNCA* expression on microglia’s immune functions during PD.

### Alpha-Synuclein’s Role in Vesicle Formation

One of the prominent functions for monomeric alpha-synuclein, primarily demonstrated in neurons, is its facilitation of synaptic vesicle formation. The protein associates with fatty acids and lipids within vesicle membranes to promote the assembly of the SNARE complex, which drives the fusion of membranes (Huang et al., 2019). In mice hippocampal neuron cultures lacking alpha-synuclein showed a reduction in the number of pre-synaptic vesicles, which correlated with lower dopamine levels in the striatum (Abeliovich et al., 2000; Cabin et al., 2002). In line with this observation, transgenic expression of alpha-synuclein rescued neurodegeneration in mice lacking CSPα, a presynaptic chaperone protein important in SNARE complex formation (Chandra et al., 2005; Burre et al., 2010). Conversely, deletion of alpha-synuclein accelerated neurodegeneration in mice (Chandra et al., 2005; Burre et al., 2010). These experiments show that monomeric alpha-synuclein is necessary for SNARE complex assembly and deficits in the protein can impact vesicle formation. Alternatively, aggregated alpha-synuclein inhibits SNARE-mediated membrane fusion (Hawk et al., 2019). The data explains a possible mechanism for impairments in SNARE-mediated neuronal dopamine release leading to neurodegeneration resulting from a lack of monomeric alpha-synuclein and/or increased aggregation of insoluble alpha-synuclein. However, as SNARE proteins are essential for a wide variety of processes involving membrane fusion, alterations in alpha-synuclein could impact vesicle formation in a broad range of cell types, especially during fetal development at the time when alpha-synuclein is most ubiquitously expressed (Ltic et al., 2004).

### Alpha-Synuclein’s Role in Vesicle Formation in Microglia

The effects of alpha-synuclein deficits or aggregation on SNARE-mediated vesicle formation in microglia have not yet been demonstrated. However, because vesicle formation during autophagy is critical for microglia to phagocytose and deliver extracellular cargo to the lysosome for degradation, the lack of endogenous alpha-synuclein could impact vesicle formation like what is observed in neurons. SNARE proteins are indeed required for the formation of precursor vesicles that give rise to the phagophore during the initiation phase of autophagy (Wang et al., 2016). This mechanism in microglia aids in the removal of toxic proteins and is linked to protection against the accumulation of neuron-derived alpha-synuclein (Choi et al., 2020). Any impairments in vesicle formation during autophagy could influence microglia’s ability to clear misfolded alpha-synuclein and thus contribute to its aggregation. Although, defective autophagy is seen in PD patients and *in vivo* studies show that impairments in microglial autophagy specifically, leads to neurodegeneration in mice, there is still a loose connection between impaired autophagy in microglia and PD in humans, with no studies investigating alpha-synuclein’s role in this paradigm (Karabiyik et al., 2017; Qin et al., 2021).

Vesicle exocytosis during cytokine release from microglia is also mediated by the SNARE complex (Murray et al., 2005). Evidence suggests that this process involves alpha-synuclein because cytokine release in microglia is impaired by both over-expression and deficient levels of alpha-synuclein in mice (Austin et al., 2006, 2011; Gardai et al., 2013). Gardai et al. (2013) data suggests that a possible mechanism leading to impaired cytokine release could be via alpha-synuclein’s interaction with SNAP23, a component of the SNARE complex. However, this was tested in the H4 neuroglioma cell line. This and other studies show there are multiple functions involving vesicle formation that are potentially facilitated by alpha-synuclein. Vesicle formation is critical for many processes in microglia that provide protection against toxic protein accumulation and to facilitate inflammatory responses in the brain. Further research is needed to determine if alpha-synuclein interacts with SNARE complexes in microglia and what the impacts of alterations in endogenous alpha-synuclein are on autophagic clearance of toxic proteins and cytokine release in microglia.

### Alpha-Synuclein’s Influence on Microglia in PFF Models

Gene-environment interactions play a significant role in PD risk (Cannon and Greenamyre, 2013). For example, as discussed above, viral infections may trigger an inflammatory event that lead to the seeding of pathological alpha-synuclein aggregates, which could be aggravated in the presence of PD-associated mutations. Most of the studies mentioned in previous sections have been done in *in vivo* and *in vitro* models that rely on transgenic or viral over-expression, which represents genetic related changes in endogenous microglial alpha-synuclein. The PFF model, however, represents an environmental trigger or extracellular source of fibrillar alpha-synuclein. In the PFF model, injection of alpha-synuclein fibrils leads to the conversion of endogenous alpha-synuclein into pathological aggregates (Volpicelli-Daley et al., 2011, 2014; Chung et al., 2020). The levels of alpha-synuclein in these models are more physiologically relevant and the spatial and temporal spread of pathology better mimics what is observed in the PD brain (Duffy et al., 2018a). Furthermore, dopamine neuronal dysfunction is observed prior to the onset of motor symptoms as is seen in PD patients (Kordower et al., 2013; Chung et al., 2020). For these reasons PFF models are considered one of the best tools for studying neurodegeneration in synucleinopathies and should be used in combination with transgenic models to understand the compounding effects of genetic and environmental risk. Indeed, it has been demonstrated that viral-mediated overexpression of alpha-synuclein in dopamine neurons exacerbates alpha-synuclein pathology and neuroinflammation in combination with PFF injection (Thakur et al., 2017). The question remains as to whether genetic variants and mutations that impact the endogenous expression of *SNCA* or alpha-synuclein function in microglia specifically, has a synergistic effect on neurodegeneration induced by exogenous alpha-synuclein in a PFF model.

Findings from PFF models alone indicate that microglia display phenotypes that influence neurodegeneration. Duffy et al. (2018b) showed a positive correlation with alpha-synuclein inclusion load and the number of MHC-II positive microglia prior to neurodegeneration in a rat PFF model, suggesting that microglia activation, in response to exogenous phosphorylated alpha-synuclein, is an early event that may contribute to neuronal vulnerability. This same phenotype has been reported in PD patients (Croisier et al., 2005). Other alpha-synuclein PFF models further demonstrate persistent microglial reactivity and cytokine release that is associated with neurodegeneration (Zhang et al., 2005; Yun et al., 2018). In marmosets injected with PFFs of alpha-synuclein, microglia also seem to play a protective role through phagocytic clearance of alpha-synuclein (Shimozawa et al., 2017). These studies demonstrate that alpha-synuclein PFF models displays robust immune responses in microglia. However, the mechanisms in which alpha-synuclein fibrils exert their effects are not well characterized. Proteomics data from Sarkar et al. (2020) showed that alpha-synuclein PFFs induce expression changes of microglial genes involved in RNA binding, mitochondrial stress, and lysosomal and autophagic function. They confirmed that some of these genes, like *Grn* which codes for progranulin, have been implicated in PD risk through GWAS. Models utilizing PFFs have thus provided novel insights into microglia behavior when exposed to toxic alpha-synuclein fibrils. The molecular pathways affected by fibrillar alpha-synuclein may converge on pathways influenced by genetic risk variants and mutations and thus dictate the severity of functional changes that increase risk for PD.

Limitations of the PFF model have posed difficulties in defining their immune responses. Processes triggered by alpha-synuclein fibrils seem to be highly dependent on the site of PFF injection, the phosphorylation status of the seed, the method of PFF preparation, the compatibility of fibrils with the host species, and the timing of injection/pathological examination (Karampetsou et al., 2017; Polinski et al., 2018; Chung et al., 2020; Chmielarz and Domanskyi, 2021), all of which may affect immune phenotypes. Furthermore, the PFF model shows a slower stepwise progression of alpha-synuclein pathology that may not lead to as exaggerated inflammatory responses comparable to what is displayed in over-expression models. These factors likely contribute to variations in the degree of pathology seen in PFF models (Karampetsou et al., 2017). Some work has focused on overcoming this issue by standardizing and publishing best methods for model generation (Polinski et al., 2018). Despite the limitations of the PFF model, it is well suited for investigating the effects of neuroinflammation on PD as it closely recapitulates the events in idiopathic PD. Moving forward alpha-synuclein PFF models will be an important tool for evaluating how PD risk genes, like *SNCA*, in microglia, function in an already established disease state or in the context of compounding risk through exposure to environmental triggers for PD.

## Concluding Remarks

Alpha-synuclein dysfunction or alterations in expression levels are one of the most well-known contributing factors to PD. These changes affect the function of neurons, especially at later stages in the disease. Genetic studies, however, indicate that alterations in *SNCA* expression occur in multiple cell types. In some cases, *SNCA* is expressed at highest levels in precursor cell populations like hematopoietic stem cells, with fluctuations in the levels of alpha-synuclein leading to observable effects on differentiation. This suggests that abnormalities in *SNCA* expression beginning from conception could dictate the composition of mature cell populations residing in the brain and their function later in life.

More recent studies have shown that alpha-synuclein influences the function of immune cells, with important implications for PD risk. For example, macrophages from PD patients appear to be affected by mutant forms, and loss or gain of alpha-synuclein levels (Haenseler et al., 2017). Microglia are the resident macrophages in the brain, and functional changes in this cell type leading to excess inflammation are associated with PD. Microglia have been extensively studied for their contribution to neurodegeneration, yet there is little known about the function of endogenous alpha-synuclein in these cells, especially during their development. As discussed above, *SNCA* is expressed at high levels in hematopoietic stem cells, with varying levels of alpha-synuclein affecting hematopoiesis of peripheral immune and blood cells. Microglia also originate from hematopoietic precursors, and as the *in vivo* data from Svoboda et al. (2019) suggest, *SNCA* expression is higher in microglia precursors compared to more mature cells. Alpha-synuclein may therefore be most active during the differentiation process in microglia. Consequently, hematopoietic dysfunction could influence microglia inflammatory responses in the brain following differentiation. The studies investigating the activity of endogenous alpha-synuclein in mature microglia do show that altered *SNCA* expression is linked to impaired cytokine release and phagocytosis (Austin et al., 2011; Grozdanov and Danzer, 2020). The evidence demonstrating that alpha-synuclein influences hematopoiesis and inflammatory responses provides precedence to explore the functions of alpha-synuclein in microglia as they develop and mature, to determine if dysfunctional differentiation and immune responses in microglia contribute to increased risk for PD.

We briefly discussed the involvement of alpha-synuclein in vesicle formation, which appears to be a mechanism that is linked to altered dopamine signaling in neurons during PD. Since this mechanism requires the involvement of alpha-synuclein in SNARE complex formation, one could speculate that alpha-synuclein also plays a role in SNARE complex formation in microglia, which is a critical step for the formation of vesicles involved in autophagic clearance of toxic proteins such as aggregated alpha-synuclein, and cytokine release. Although there is little evidence of genetic linked alterations in *SNCA* expression and alpha-synuclein function in microglia leading to changes in vesicle-mediated processes such as autophagy, it is worth exploring as a possible mechanism leading to alpha-synuclein aggregation and neurodegeneration in PD.

It is difficult to dissect the influence of certain cell populations early on in disease. However, models using PFFs of alpha-synuclein and iPSCs derived from PD patients are beginning to give us clues to the changes that occur in PD patients prior to the onset of neurodegeneration. Understanding the early biological processes involving imbalances in alpha-synuclein expression in cell types like hematopoietic stem cells and microglia, will contribute to the understanding of the influence of genetics on PD risk and may provide avenues for therapeutic intervention before PD manifests into motor impairment.

## Author Contributions

AB designed and drafted all sections of the manuscript and performed all data analysis and prepared the figures. GC contributed to manuscript design and review. Both authors contributed to the article and approved the submitted version.

## Conflict of Interest

The authors declare that the research was conducted in the absence of any commercial or financial relationships that could be construed as a potential conflict of interest.

## Publisher’s Note

All claims expressed in this article are solely those of the authors and do not necessarily represent those of their affiliated organizations, or those of the publisher, the editors and the reviewers. Any product that may be evaluated in this article, or claim that may be made by its manufacturer, is not guaranteed or endorsed by the publisher.

## Supplementary Material

Supplementary Table 1 PD risk SNPs at the SNCA locus. The SNPs listed in this table were determined to be associated with PD risk by Nalls et al. (2019). The one that we believe to be relevant to microglia function, based on its location in open regulatory DNA (marked using ATAC- and H3K27ac-seq; Booms et al., 2020), is highlighted in yellow. IUPAC codes are listed at the bottom.
