# Supplementary material for: Functions of Intracellular Alpha-Synuclein in Microglia: Implications for Parkinson’s Disease Risk
Source: Front Cell Neurosci. 2021 Oct 4;15:759571. doi: 10.3389/fncel.2021.759571 (PMC8521067; doi:10.3389/fncel.2021.759571)
Supplement: Supplementary Table 1 — PD risk SNPs at the SNCA locus. The SNPs listed in this table were determined to be associated with PD risk by Nalls et al. (2019). The one that we believe to be relevant to microglia function, based on its location in open regulatory DNA (marked using ATAC- and H3K27ac-seq; Booms et al., 2020), is highlighted in yellow. IUPAC codes are listed at the bottom. [file Table_1.DOCX]

| Variation ID | Chr | Position  (GRCh37) | REF Allele | ALT Allele (IUPAC) | Minor Allele | Minor Allele Global Frequency |
| --- | --- | --- | --- | --- | --- | --- |
| rs356183 | 4 | 90626098 | G | C | C | 0.436502 |
| rs356182 | 4 | 90626111 | G | A | G | 0.404353 |
| rs356181 | 4 | 90626139 | G | A | A | 0.414337 |
| rs356180 | 4 | 90628127 | A | G | A | 0.137181 |
| rs356179 | 4 | 90628439 | C | G | C | 0.175519 |
| rs356178 | 4 | 90629465 | G | Y | G | 0.308307 |
| rs356177 | 4 | 90630675 | C | R | C | 0.121805 |
| rs796636861 | 4 | 90630801 | GTC | CTG | None | None |
| rs356176 | 4 | 90630801 | G | C | G | 0.201677 |
| rs356175 | 4 | 90630814 | C | T | C | 0.202077 |
| rs356174 | 4 | 90630901 | G | T | G | 0.202077 |
| rs356173 | 4 | 90631716 | G | A | G | 0.201877 |
| rs356172 | 4 | 90631986 | C | T | C | 0.201877 |
| rs356171 | 4 | 90632023 | C | T | C | 0.201877 |
| rs356170 | 4 | 90632276 | G | Y | G | 0.167332 |
| rs356169 | 4 | 90632768 | G | T | G | 0.201877 |
| rs2572322 | 4 | 90634546 | G | M | G | 0.169329 |
| rs2572323 | 4 | 90634552 | A | G | A | 0.169329 |
| rs6825421 | 4 | 90634806 | T | M | T | 0.169329 |
| rs6848708 | 4 | 90634828 | G | T | G | 0.169129 |
| rs181489 | 4 | 90635020 | T | M | T | 0.169329 |
| rs181490 | 4 | 90635033 | A | G | A | 0.169529 |
| rs356206 | 4 | 90635049 | T | C | T | 0.167133 |
| rs356207 | 4 | 90635407 | C | T | C | 0.169529 |
| rs356208 | 4 | 90635453 | A | T | A | 0.169529 |
| rs356210 | 4 | 90636193 | T | M | T | 0.169529 |
| rs356211 | 4 | 90636418 | C | D | T | 0.370807 |
| rs356213 | 4 | 90636489 | G | T | G | 0.245407 |
| rs356214 | 4 | 90636541 | C | T | C | 0.169529 |
| rs356215 | 4 | 90636561 | G | A | A | 0.45607 |
| rs4437213 | 4 | 90636629 | T | A | T | 0.168331 |
| rs796875394 | 4 | 90636629 | TG | AA | None | None |
| rs5019538 | 4 | 90636630 | G | A | G | 0.168331 |
| rs356218 | 4 | 90637010 | A | K | A | 0.202676 |
| rs356219 | 4 | 90637601 | G | A | A | 0.489217 |
| rs973918961 | 4 | 90641340 | T | - | None | None |
| rs375850534 | 4 | 90641340 | - | Y | None | None |
| rs356220 | 4 | 90641340 | T | M | C | 0.483427 |
| rs356221 | 4 | 90642464 | A | T | T | 0.389377 |
| rs356222 | 4 | 90643123 | C | T | C | 0.132788 |
| rs168552 | 4 | 90643144 | C | T | C | 0.107228 |
| rs202162556 | 4 | 90643623 | - | GT | None | None |
| rs356224 | 4 | 90643623 | A | K | A | 0.136581 |
| rs356225 | 4 | 90643757 | C | K | G | 0.389177 |
| rs356166 | 4 | 90649290 | C | R | C | 0.107228 |
| rs356205 | 4 | 90657186 | C | T | C | 0.1252 |
| rs356204 | 4 | 90663542 | T | C | C | 0.389377 |
| rs356203 | 4 | 90666041 | C | T | T | 0.483227 |
| rs356202 | 4 | 90666291 | G | A | G | 0.13778 |
| rs356200 | 4 | 90668614 | T | C | C | 0.389177 |
| rs189596 | 4 | 90671336 | G | M | A | 0.393371 |
| rs2736991 | 4 | 90671670 | A | Y | A | 0.108027 |
| rs356167 | 4 | 90673770 | A | S | C | 0.258986 |
| rs356168 | 4 | 90674431 | G | A | A | 0.39357 |
| rs11097231 | 4 | 90677148 | T | M | T | 0.121406 |
| rs2736990 | 4 | 90678541 | G | W | A | 0.393371 |
| rs1168111559 | 4 | 90678541 | GTGTATATGTGT | - | None | None |
| rs2572324 | 4 | 90678798 | G | A | G | 0.1248 |
| rs2737033 | 4 | 90707947 | T | S | C | 0.135783 |
| rs2583958 | 4 | 90720365 | T | C | C | 0.108027 |
| rs2737024 | 4 | 90721560 | A | G | G | 0.107827 |
| rs2583959 | 4 | 90721637 | C | K | G | 0.108027 |
| rs2619373 | 4 | 90722433 | G | A | A | 0.108027 |
| rs2619370 | 4 | 90736585 | C | W | T | 0.109625 |
| rs2619371 | 4 | 90736678 | A | G | G | 0.108227 |
| rs2737023 | 4 | 90739505 | T | C | C | 0.108227 |
| rs2737022 | 4 | 90739662 | A | C | C | 0.108227 |
| rs2583965 | 4 | 90740103 | G | T | T | 0.108227 |
| rs2583966 | 4 | 90741519 | G | M | A | 0.108227 |
| rs2583967 | 4 | 90744100 | C | T | T | 0.108626 |
| rs2619342 | 4 | 90744170 | A | T | T | 0.108027 |
| rs200874698 | 4 | 90744170 | A | - | None | None |
| rs2619343 | 4 | 90744221 | C | T | T | 0.108227 |
| rs2619344 | 4 | 90744248 | C | T | T | 0.115615 |
| rs2619345 | 4 | 90744270 | A | G | G | 0.108227 |
| rs2737019 | 4 | 90744903 | G | T | T | 0.108027 |
| rs2737018 | 4 | 90745091 | T | C | C | 0.107029 |
| rs2737017 | 4 | 90745238 | C | T | T | 0.107428 |
| rs2737016 | 4 | 90745242 | T | C | C | 0.107428 |
| rs2737015 | 4 | 90745283 | T | C | C | 0.107428 |
| rs2737014 | 4 | 90745456 | A | C | C | 0.108427 |
| rs2737013 | 4 | 90745503 | C | T | T | 0.107428 |
| rs2737012 | 4 | 90745707 | G | A | A | 0.107428 |
| rs2583969 | 4 | 90746133 | T | C | C | 0.107428 |
| rs2619348 | 4 | 90746321 | C | G | G | 0.107428 |
| rs2583970 | 4 | 90746610 | G | A | A | 0.107428 |
| rs2737009 | 4 | 90746836 | A | G | G | 0.107428 |
| rs2619350 | 4 | 90747004 | A | G | G | 0.107428 |
| rs2737008 | 4 | 90747183 | G | A | A | 0.107428 |
| rs2583971 | 4 | 90747207 | C | T | T | 0.107428 |
| rs2583973 | 4 | 90747499 | C | T | T | 0.107628 |
| rs2619351 | 4 | 90747704 | A | K | G | 0.107428 |
| rs2619352 | 4 | 90747709 | C | T | T | 0.107428 |
| rs2619353 | 4 | 90747975 | G | C | C | 0.107428 |
| rs2619354 | 4 | 90748284 | C | T | T | 0.107628 |
| rs2583974 | 4 | 90748297 | T | C | C | 0.107428 |
| rs1442146 | 4 | 90748646 | C | K | G | 0.107428 |
| rs1442147 | 4 | 90748738 | C | T | T | 0.107827 |
| rs1442149 | 4 | 90749132 | A | T | T | 0.107428 |
| rs2737006 | 4 | 90749686 | A | G | G | 0.107628 |
| rs33965306 | 4 | 90750043 | A | G | G | 0.107827 |
| rs13142587 | 4 | 90750169 | G | T | T | 0.107428 |
| rs2583976 | 4 | 90750173 | T | C | C | 0.107428 |
| rs2583977 | 4 | 90750225 | C | T | T | 0.107628 |
| rs2737005 | 4 | 90750590 | A | G | G | 0.107428 |
| rs2737004 | 4 | 90750722 | A | G | G | 0.107428 |
| rs2583980 | 4 | 90751109 | T | G | G | 0.107628 |
| rs986609 | 4 | 90751415 | C | K | G | 0.107428 |
| rs986610 | 4 | 90751655 | T | C | C | 0.107428 |
| rs2583981 | 4 | 90752582 | A | T | T | 0.107628 |
| rs2619358 | 4 | 90752670 | A | C | C | 0.107428 |
| rs2737003 | 4 | 90753068 | A | G | G | 0.107428 |
| rs2583982 | 4 | 90753244 | T | C | C | 0.107428 |
| rs2737001 | 4 | 90753477 | G | A | A | 0.107428 |
| rs2583983 | 4 | 90753960 | C | T | T | 0.107827 |
| rs2737000 | 4 | 90754022 | C | A | A | 0.107428 |
| rs2736999 | 4 | 90754111 | A | G | G | 0.107428 |
| rs2583984 | 4 | 90754153 | T | C | C | 0.107628 |
| rs2736998 | 4 | 90754336 | C | A | A | 0.107428 |
| rs2736997 | 4 | 90754557 | G | A | A | 0.107428 |
| rs2736996 | 4 | 90754691 | A | G | G | 0.107428 |
| rs2619359 | 4 | 90754828 | A | C | C | 0.107428 |
| rs990086 | 4 | 90755090 | A | T | T | 0.107428 |
| rs990087 | 4 | 90755177 | A | C | C | 0.107428 |
| rs990088 | 4 | 90755225 | T | C | C | 0.107428 |
| rs1372516 | 4 | 90755730 | G | A | A | 0.107428 |
| rs2583985 | 4 | 90755939 | A | G | G | 0.107428 |
| rs2619360 | 4 | 90756191 | C | T | T | 0.107628 |
| rs2028535 | 4 | 90756421 | G | C | C | 0.107428 |
| rs2619361 | 4 | 90757735 | C | A | A | 0.107827 |
| rs2619362 | 4 | 90757845 | C | W | T | 0.107827 |
| rs2619363 | 4 | 90759047 | G | Y | T | 0.107428 |

| IUPAC Code | Meaning |
| --- | --- |
| G | G |
| A | A |
| T | T |
| C | C |
| R | G or A |
| Y | T or C |
| M | A or C |
| K | G or T |
| S | G or C |
| W | A or T |
| H | A or C or T |
| B | G or T or C |
| V | G or C or A |
| D | G or A or T |
| N | G or A or T or C |
